# Case of Hemorrhage and Abortion

**Published:** 1867-07

**Authors:** D. B. Trimble

**Affiliations:** Chicago


ARTICLE XXX.

### CASE OF HEMORRHAGE AND ABORTION.

By D. B. TRIMBLE, M.D., Chicago.

I was called, on the night of March 18th, to attend a lady on the West Side, and found her suffering from acute spasmodic action of the womb, accompanied by severe uterine hemorrhage. I was informed that she had had a convulsion, but this had passed away before my arrival. On the evening of the 13th, while sitting with her feet elevated on a stool or chair, writing, she had a sudden gush of blood from the uterus, unaccompanied by pain. The hemorrhage continued at intervals, and the pains increased, but, her husband being absent from the city, I was not sent for until the night above mentioned. In reply to the question whether her catamenia had been regular, I was assured that they had been. The discharge was very profuse, and her suffering, though abated, was severe. Her pulse, however, was not much depressed. Prescribed the mineral acids, and a solution of morphine, and locally, a strong and cold solution of Alum; the recumbent position, and perfect rest

On the *19th*, considerably relieved; hemorrhage lessened.

*21st.* So much better that I thought it unnecessary to see her again, and recommending a continuance of the recumbent posture, and, to some extent, the medicines, for a few days, I left her.

On Saturday, the *23d*, she took a walk, which brought on a return of the hemorrhage, and on Monday, *25th*, I was again sent for, and found her suffering very acutely, and flooding severely.

From her husband, who was now at home, I obtained a history of the case. In December last, she had passed her regular menstrual period by about ten days, when she became anxious about her condition, and wished the catamenia restored. She had had one child, and, by her own account, after a very difficult and dangerous labor, and therefore wished to avoid a recurrence of the same trouble. Her husband proposed sending for me, but she objected, on the ground that I would probably not do for her what she desired (and this supposition was correct). She therefore sent for a neighboring homœopath, who gave her a preparation of savin, which, in the course of a week, brought on severe hemorrhage and suffering, and she supposed she was relieved of her burthen. Every three weeks, subsequently, until I saw her, she had a return of the menstrua in great and increasing abundance, and with increased suffering. This fact was a further confirmation of her views, that she was not pregnant, and this circumstance made me, also, incline to the same opinion; but, from the continued firmness of her pulse, the liquid character of the discharge and its abundance, and the severity of her pains, I was inclined to the opinion that, if not caused by pregnancy, there was probably a polypoid or other tumor in the womb. I therefore requested permission to make a tactile examination; but there was no evidence of any tumor; the os uteri was in a normal condition, closed, and not tender. This theory was, therefore, not confirmed, though not certainly disproved.

On the *26th*, under the above-mentioned and other remedies, she again appeared much relieved, and I did not see her again until the *28th*, when she was still apparently improving.

On the *29th*, though the hemmorrhage still continued to some extent, she was comfortable; but on the night of the *29th*, I was again called, when I found her deluged with blood; suffering much; paler, but still with a pulse of much force. The heart's action, however, began to be affected, and, at times, there was considerable intermission in its pulsations. I now resorted to the tampon, saturated with alum water, which appeared to control the hemorrhage for that night and the next day.

On the *30th*, found her comfortable, cheerful, and without hemorrhage. In the course of this week, had given her a combination of alum and nutmeg (a favorite remedy of Dr. CHAS. D. MEIGS), fld. ext. ergot, etc.

On the *31st*, comparatively comfortable; but some return of hemorrhage. Removed the tampon, and introduced a very soft sponge, saturated with a solution of tannic acid. Hemorrhage continued, however, but the blood was now clotted- Upon re-examination, found the os more tumefied and tender, and the uterus itself enlarged and lower; and placing my hand over the pubic region, found the uterus considerably enlarged and firm. My diagnosis was still uncertain. Was the enlargement of the womb owing to pregnancy, to a tumor, or to coagulated blood accumulating in the womb, and retained by its rigid mouth? In my difficulty, I laid the case before an experienced medical friend, who thought, with me, that it was very improbable she was pregnant, and suggested that, from the increasing tenderness and enlargement, it might be sub-acute hysteritis.

*April 1st and 2d.* Quiet; hemorrhage and pain sometimes nearly ceasing, then returning, but not so freely.

*3d.* Took with me a strong sol. of ext. atrop. belladon., with the intention of applying it to the rigid os tincæ, so as to produce dilation; and when this was effected, it would probably enable me to determine the cause, whether from coagula, a tumor, or a fœtus; and, if proper, I would then administer the ergot until the uterine contractions were sufficient to expel its contents. I called at 11 o'clock A.M., and found her so comfortable, and enjoying the company of a lady friend, that I delayed its application until my next visit. At 2 o'clock P.M., however, I was sent for in great haste, with the assertion that she was flooding to death. I found her in great pain, though intermitting; on examination per vagina, that there was but little hemorrhage, and that the os was slightly dilated. Saturated the sponge with sol. belladon. and reapplied it. Seeing the great anxiety of the husband, and, in fact, being anxious myself, I requested that Dr. JONES (of Peoria Street), who lived near by, should be sent for. By the time he had arrived, the os had become sufficiently dilated to admit the insertion of a finger, and, after a careful examination, Dr. Jones believed there was a fœtus; and, after again examining her, I no longer felt any doubt. The doctor concurred in my proposal to use the belladonna and ergot, and left me. This was about $3\frac{1}{2}$ o'clock P.M. I at once administered f℥ss. of the ergot, and reapplied the belladonna. Every half-hour, I administered the former, until she had taken f℥ 4 or 5. The pains became more regular and urgent; but still the os relaxed very slowly. It was a breech presentation, and after frequent and prolonged efforts, the body and limbs were born, but the head remained, and the pains ceased. After waiting ten or fifteen minutes, I made an effort to renew the contractions and bring away the head, but owing to the rigid condition of the os uteri, (which contracted around the neck,) I could introduce but one finger. I made gentle traction by the body, but very carefully, fearing a separation of the head. After a time, what I feared, actually occurred, and introducing my finger, found the head had receded so that I could not reach it. I now introduced a pair of small abortion forceps, and though I could touch the head, could not grasp it. I then grasped the placenta, and, finding that it did not adhere, removed it entire. After again making efforts to get the head between the blades of the forceps, without success, I gradually dilated the os uteri until I could get two fingers into it, and passing them up until I reached the head, bent them over it, and brought it away. The patient recuperated more rapidly than I expected, and is now well.

When we take into consideration the apparently successful effort to produce abortion in December; and the regular, though frequent and profuse return of the menstrua, up to March 13th; and the absence of the general signs of pregnancy (which had been apparent in her first); I think I am excusable in failing in my diagnosis. I have occasionally known persons to have a slight monthly discharge during pregnancy, sometimes to the period of parturition, but never before, so great a discharge, without destroying and dislodging the fœtus.

